# COVID-19 Vaccine Hesitancy: Analysing Twitter to Identify Barriers to Vaccination in a Low Uptake Region of the UK

**DOI:** 10.3389/fdgth.2021.804855

**Published:** 2022-01-24

**Authors:** Katherine Lanyi, Rhiannon Green, Dawn Craig, Christopher Marshall

**Affiliations:** National Institute for Health Research Innovation Observatory (NIHR) Innovation Observatory, Population Health Sciences Institute, Newcastle University, Newcastle, United Kingdom

**Keywords:** COVID-19 vaccination, tweets, topic clustering, artificial intelligence (AI), natural language processing (NLP), vaccine hesitancy, geo-location

## Abstract

To facilitate effective targeted COVID-19 vaccination strategies, it is important to understand reasons for vaccine hesitancy where uptake is low. Artificial intelligence (AI) techniques offer an opportunity for real-time analysis of public attitudes, sentiments, and key discussion topics from sources of soft-intelligence, including social media data. In this work, we explore the value of soft-intelligence, leveraged using AI, as an evidence source to support public health research. As a case study, we deployed a natural language processing (NLP) platform to rapidly identify and analyse key barriers to vaccine uptake from a collection of geo-located tweets from London, UK. We developed a search strategy to capture COVID-19 vaccine related tweets, identifying 91,473 tweets between 30 November 2020 and 15 August 2021. The platform's algorithm clustered tweets according to their topic and sentiment, from which we extracted 913 tweets from the top 12 negative sentiment topic clusters. These tweets were extracted for further qualitative analysis. We identified safety concerns; mistrust of government and pharmaceutical companies; and accessibility issues as key barriers limiting vaccine uptake. Our analysis also revealed widespread sharing of vaccine misinformation amongst Twitter users. This study further demonstrates that there is promising utility for using off-the-shelf NLP tools to leverage insights from social media data to support public health research. Future work to examine where this type of work might be integrated as part of a mixed-methods research approach to support local and national decision making is suggested.

## Introduction

The global COVID-19 pandemic is the most significant healthcare emergency in recent memory, creating an unprecedented burden on healthcare systems (1). Since the first case was reported on 31 January 2020, there has been over 7 million cases, and to date almost 135,000 deaths associated with SARS-CoV-2 in the UK (2, 3). Globally, rapid progress was made to develop highly effective vaccines that could reduce transmission and burden of disease. At the time of data-collection, there were 3 vaccines approved for use in the UK by the Medicines and Healthcare Regulatory Agency (MHRA) (4, 5). In order to realise the potential that vaccines offer as a route out of the pandemic to a more endemic state with lower rates of severe disease, high rates of vaccination must be achieved (6). Whilst opinion varies as to the percentage uptake necessary to achieve “herd immunity,” some experts suggest it could be as high as 95% (7).

The UK vaccination programme commenced on 08 December 2020. As of 15 August 2021, every adult UK resident had been offered the first dose of a MHRA-approved vaccine. Initially, public enthusiasm to receive a COVID-19 vaccine was high, with supply—not demand—being the limiting factor (6, 8). This enthusiasm continued until the start of July 2021 when a plateau in the culminative number of vaccines administered was observed (9). As of 15 August 2021, 89.3% of those eligible in the UK had received their first dose (8). However, significant regional variation in vaccination rates was (and remains) apparent across different regions of the country. In London, for example, only 82.3% of residents eligible for a vaccine had received a first dose by the same date (August 2021) (9, 10). As demonstrated by previous immunisation campaigns for other transmissible diseases (e.g., winter flu and meningococcal serogroup C), greater uptake can be achieved through targeted intervention strategies addressing specific barriers to vaccination (11, 12). For interventions to be appropriately targeted, the reasons underpinning vaccine hesitancy in low-uptake areas must be clearly understood. Traditional methods of collecting data relating to public opinion, such as surveys or focus groups, are time, money, and resource intensive and faced particular challenges in the current pandemic climate (13). Further in a fast-moving situation, such as the pandemic, it remains unclear whether traditional methods offer a more robust solution than a more rapid, pragmatic approach to gaining insight and facilitating rapid development and delivery of interventions that are crucial (14, 15).

More than 77% of the UK population are active on social media (e.g., Twitter, Facebook, Reddit), and with usage increasing during the pandemic, vast amounts of rich data exist within these so called “soft-intelligence” sources (16, 17). Novel approaches capable of extracting and analysing these data to provide actionable insight into public perceptions have the potential to transform the landscape of public health research (15, 18, 19). In addition to the increased volume of data that soft-intelligence sources provide, user generated content on social media is freely volunteered and not restricted to the scope of the question being posed (13). Previous studies have shown that when leveraged using artificial intelligence (AI)-based techniques including NLP, insights from social media can be useful to rapidly understand public opinion, sentiment, and behaviour (20–22).

The aim of this work was to further investigate and explore the value of using insight gained from social media, as a meaningful source of intelligence to support public health research. In this article, we report the findings from a short case study, in which we deployed a NLP platform to rapidly detect and analyse key barriers to vaccine-uptake from a sample of geo-located tweets posted from users in the region of London, UK.

## Methods

### Search and Data Collection Strategy

We selected Twitter as our chosen data source for collection and analysis. Twitter is a social media platform where users can post short messages “tweets” up to 280 characters long. Twitter is amongst the most popular social media platforms in the UK with over 17.5 million active users, as of July 2021 (23). Twitter data has been used successfully in previous surveillance studies as a source of real-time user-generated data to track trends in public dialog and perceptions over time and recognise what is happening on the ground during a viral pandemic (20, 24, 25).

We developed a search strategy comprising the following list of terms:

(vaccine OR vaccines OR vaccinate OR vaccinates OR vaccination OR vaccinations OR vaccinated OR vax OR vax OR anti-vax OR anti-vaxx OR antivax OR antivaxx) OR (covid OR coronavirus) (moderna OR pfizer OR BioNTech OR AstraZeneca)

This strategy was used to search for any relevant geo-located tweets posted by users in London, UK relating to COVID-19 vaccines or the vaccine roll-out. The specific search terms and syntax were generated through discussion between members of the National Institute for Health Research (NIHR) Innovation Observatory soft-intelligence and information research working groups, along with scanning relevant literature, including recently published studies and news articles (25–27).

Once the search strategy was confirmed, we began prospectively searching for and collecting relevant tweets via Twitter's advanced search application interface (28). The search ran over an 8-month period, between 30 November 2020 and 15 August 2021. This time-period covered the approval of the first vaccine in the UK through to all UK citizens aged 18 years and over being offered the first dose (9). All tweets identified in the search were anonymised to protect the privacy of users.

### Data Analysis

#### Natural Language Processing

An advanced AI-based, text analytics platform using NLP was used to initially analyse the tweets. The analytics platform, “Wordnerds,” is described by its developers as a “text analysis and insights platform using machine learning techniques” (29). In particular, this off-the-shelf platform supports analysis of meta-data, topic, and sentiment to understand the context of a tweet and group tweets together into topic clusters that contains tweets relating to each other, or discussing similar issues. This facilitates a more accurate and sophisticated insight in to the vaccine conversation on Twitter compared to methodologies which rely solely on a qualitative count of single words, phrases, or hashtags (30).

For this study, we used the platform to analyse the volume and sentiment of the collated tweets, and to identify key topics of negative discussion within the dataset. On loading the tweets, the platform was able to determine the sentiment of each tweet and then clustered them accordingly with others that discussed the same (positive or negative) topic.

Based on the analysis of the initial corpus of tweets, the platform automatically generated 12 clustered topics of conversation related to COVID-19 vaccination with negative sentiment. The clustered topics included the following:

1) “covid vaccine,” 2) “vaccine passports,” 3) “people vaccinated,” 4) “worry vaccine,” 5) “vaccines work,” 6) “vaccine rolled,” 7) “second dose,” 8) “having vaccine,” 9) “thing vaccines,” 10) “booking vaccine,” 11) “coronavirus vaccine” and 12) “az developed vaccine.”

The tweets contained within these 12 topic clusters were used as our sample to ascertain potential barriers to vaccine hesitancy via qualitative document analysis.

#### Coding Tweets to Hesitancy Themes

Using the sample set generated by the platform, tweets were manually coded by 2 researchers (KL and RG), independently, to one of 6 pre-determined themes relating to vaccine hesitancy:

1) Mistrust, 2) Safety, 3) Ineffective, 4) Access, 5) Under-representation, or 6) Complacency.

These themes were developed based on the Scientific Advisory Group for Emergencies (SAGE) working group's 3C model of vaccine hesitancy, published by the World Health Organisation (31). This model establishes three core barriers that determine levels of vaccine uptake: confidence (i.e., the level of trust in the efficacy and safety of the vaccine), complacency (i.e., the perceived need for the vaccine), and convenience (i.e., how accessible the vaccine is to people) (31, 32). The 3C model emphasises that whilst all vaccine hesitancy is grounded in “the 3Cs,” the specific issues underpinning these core barriers (such as safety, efficacy, cost and trust) are context specific to a particular vaccine, and the circumstances for which it is being developed (31, 33).

Following close examination and consideration of the 3C model, and based on our study's context and target population, we arrived at the 6 tailored themes listed above. We note that the themes we have selected do not cover all those considered by the 3C model. For example, affordability, an important reason underpinning the 3C's model convenience barrier, was not included for this study, since vaccines are freely-available to the UK public via the National Health Service (NHS). London has a relatively high population of ethnic-minority residents who, according to polling, are least likely to get vaccinated (27, 34, 35). Therefore, we also included “under-representation” as a vaccine hesitancy theme for our analysis.

Whilst mapping tweets to one of our six vaccine hesitancy themes, tweets deemed to be posting false content (i.e., misinformation) about vaccines were also tagged by the researchers. A tweet was coded and tagged as misinformation if the content it shared had not been verified from reputable sources.

#### Qualitative Document Analysis

Qualitative document analysis is an established methodological approach to synthesise printed and electronic materials (36). This technique has been used in previous work for exploring and analysing social media datasets to provide intelligence for public health surveillance (21, 37, 38). Two researchers (KL and RG) independently undertook qualitative document analysis of the sample set of tweets automatically generated by the platform to provide more insight, contextualise the results, and strengthen the overall analysis. A third, senior researcher (CM) sense checked the results. The findings were discussed by all authors to form a consolidated final set.

## Results

### Search Results and Included Tweets

**Figure 1:**
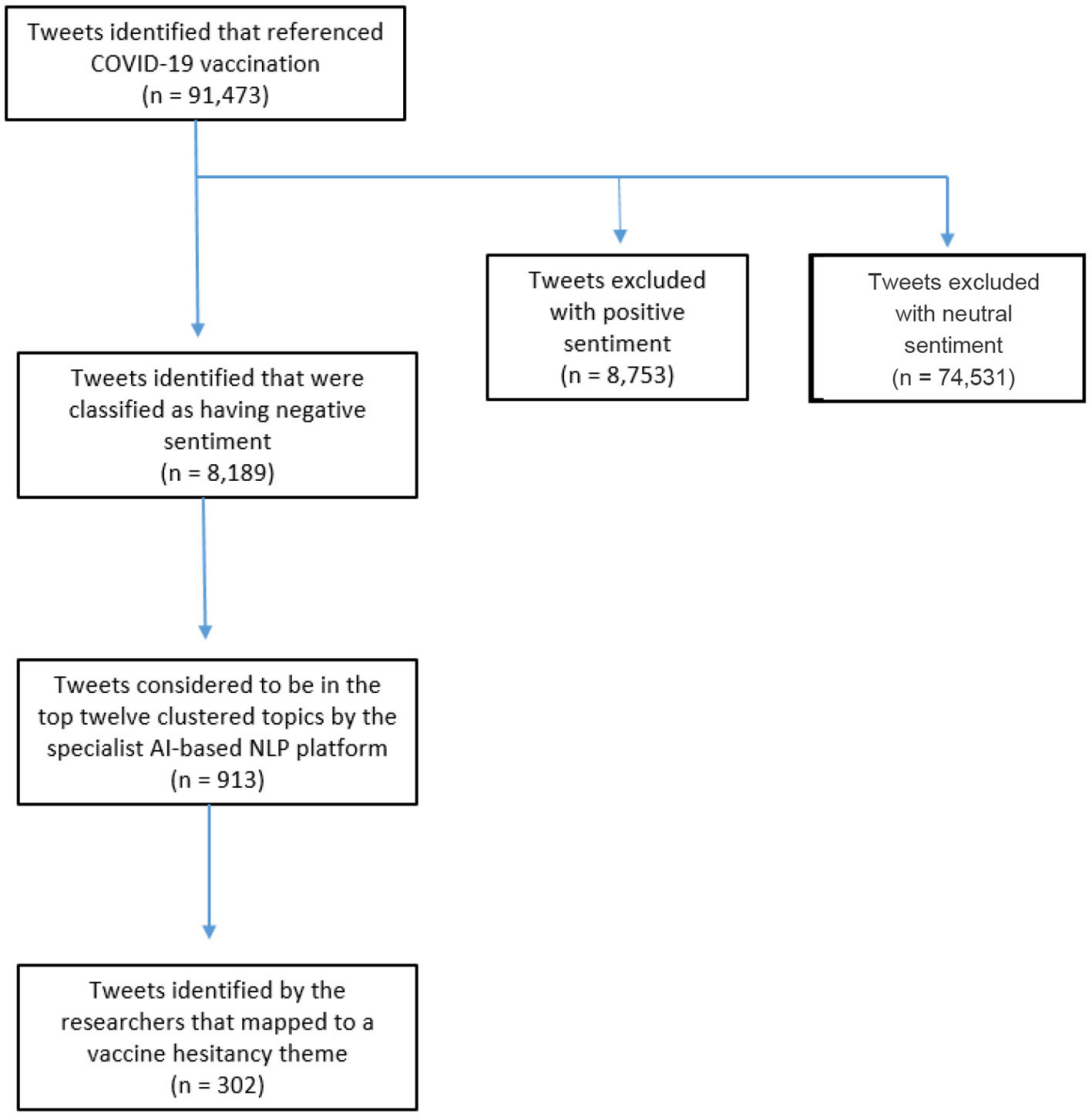
Search strategy and Tweet selection flow diagram.

The search strategy identified 91,473 initially relevant tweets. Of these, 82,284 were excluded due to the NLP tool classifying these tweets as having either positive or neutral underlying sentiment. The remaining 8,189 (9%) classified as having negative sentiment were fed through the NLP tool's topic analysis algorithm, which generated 12 clustered topics of discussion based on 913 of those tweets. The tweets contained within these 12 clusters represented ~1% of the tweets scraped in the initial search strategy and 11% of those classified as having negative sentiment. Figure 1 visualises the flow of tweets from the initial search to the final sample used in the analysis.

**Figure 2:**
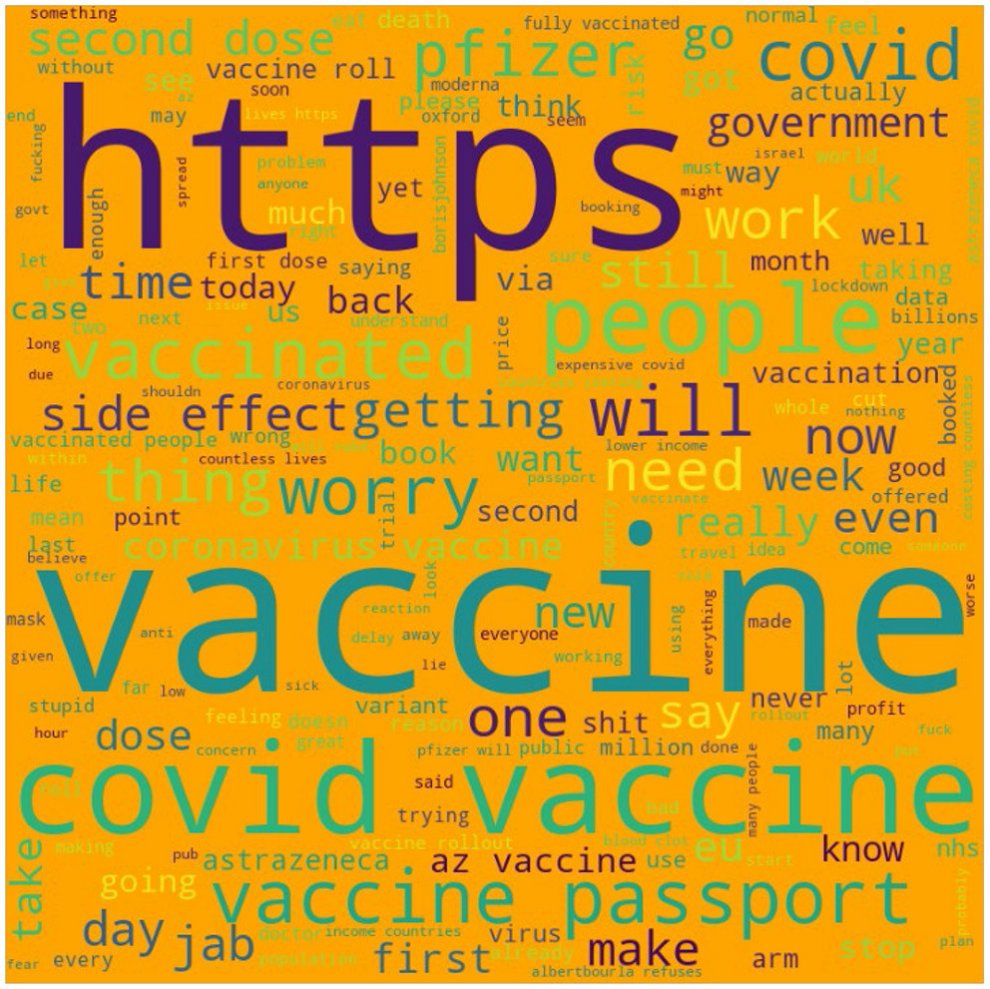
Visual representation of the most commonly discussed topics within the dataset.

Separately from the platform we used to carry out the main analysis, we wrote some code in Python to visually represent the most commonly discussed topics within the 913 sample tweet using the “wordcloud” package (see Figure 2) (39). Topics that appeared most often were the most significant drivers of negative vaccine sentiment. The larger the word appears on this figure, the greater frequency that word was mentioned in the sample. “https” is one of the most mentioned words due to many people sharing (mostly inaccurate) links relating to the vaccine.

### Coding Tweets to Hesitancy Themes

On manually screening the sample of 913 tweets, 302 (33.1%) provided enough detail to contextualise the negative sentiment and subsequently code to a hesitancy theme. 611 tweets were not considered eligible for mapping. This was mainly due to the tweets negatively discussing other political/societal factors surrounding the vaccines (e.g., anger towards proposals to delay the second dose, vaccine passports etc.) rather than users expressing these issues were a barrier to them receiving a vaccination.

**Figure 3:**
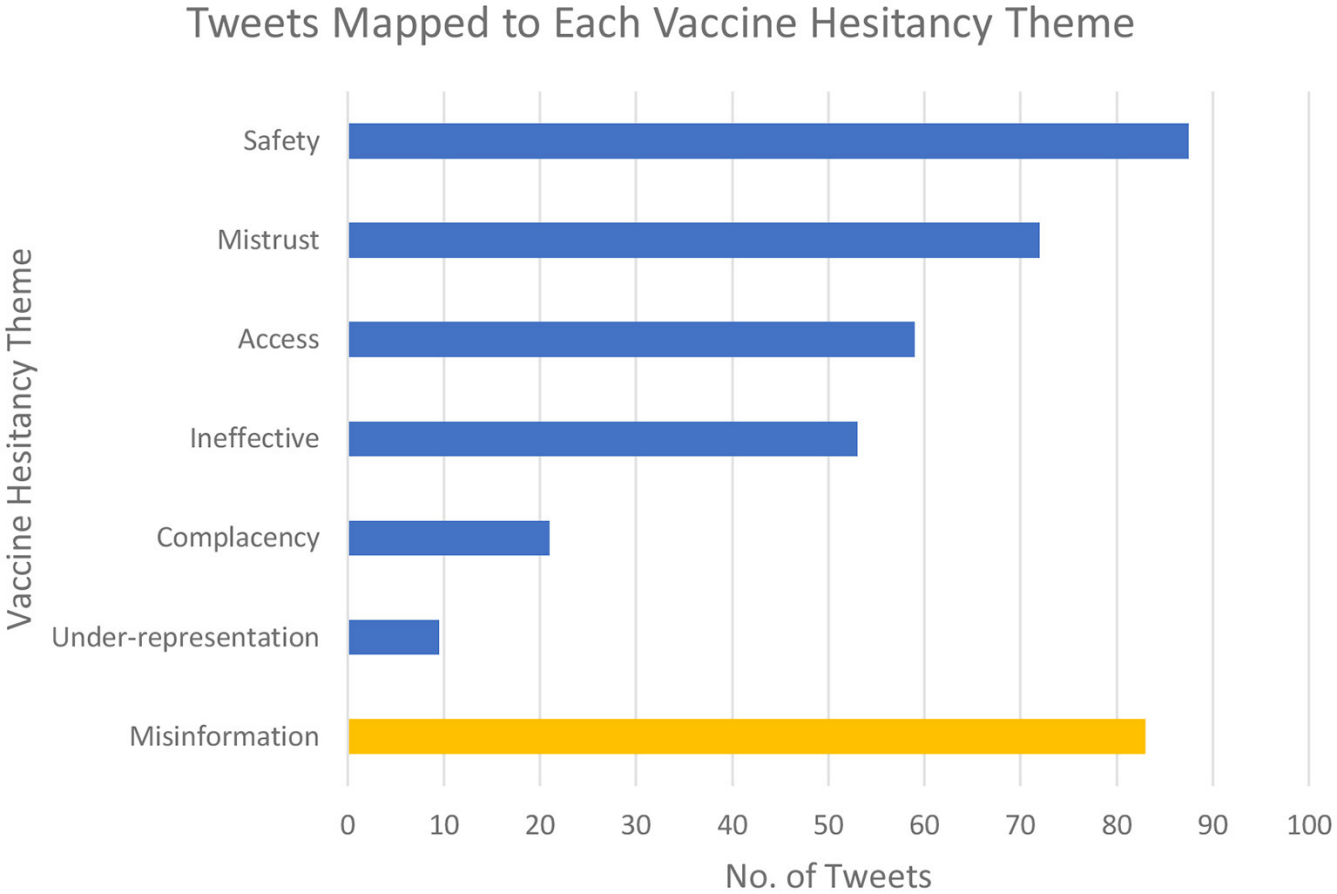
Chart to quantify the results of the coding exercise.

More tweets were coded to the safety and then mistrust themes than any other; with 88 (29%) and 72 (23%) coded, respectively. Under-representation was the least mapped theme accounting for only 10 (3%) of coded tweets. 83 tweets (just under 10% of the initial sample) were identified as tweets that contained misinformation (see Figure 3).

### Results of Qualitative Document Analysis

In this section, the results of the qualitative document analysis of tweets that were mapped to each theme are presented. Selected example tweets that were mapped are presented in Table 1.

**Table 1:** Exemplar tweets mapped to each theme.

**Mapped barrier**	**Selected example tweets**
Safety	“*I'm getting my Covid vaccine tomorrow however, I'm scared it'll affect fertility 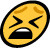 ”* “*Nothing at all to worry about… Pfizer Covid vaccine: Side effects may include pulmonary embolism |”*
Mistrust	“*I'm sorry, but if @RishiSunak is found to have been making personal profits from investing in COVID vaccine companies, he needs to be sacked. Does conflict of interest have no meaning to the @Conservatives ? So, so corrupt.”* “*Spot on variants and vaccine passports are the threat to pushing this vaccine, it all started off for the old and vulnerable and protecting the nhs, now cancer and heart trouble have taken over, this covid charade is a smoke screen.”*
Access	“*All I want is a chance to at least book an appointment for a vaccine, but here I am trying to learn code and sign up to bots to get a slot”* “*I have received 2 messages regarding COVID vaccine but with all the scammers I can't even be sure if this is valid 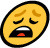 ”*
Ineffective	“*If antibody levels are this high, yet we are still seeing so many “cases” surely this is now just endemic? The vaccines don't prevent you getting or transmitting so what's the point of vaccine passports when vaccination rates are touching 90%”* “*Why are there still more deaths in the 80+ age group than any other, given this most vulnerable group was vaccinated first? There is a clear inconsistency here, either the vaccine does not work, or COVID is not the cause of their death.”*
Complacency	“*I tired hearing bout COVID, curfew, vaccines and all that other cxnt”* “*I'm not a fan of national sheep training (aka football) but vaccine passports are just another bungle during the great covid over reaction”*
Under-representation	“*Black folk still need to take the COVID vaccine but I honestly have no time for White ppl blowing the anti-vaxer horn when Black ppl share their fears. This isn't the same conversation as your middle class White mum who just discovered essential oils & herbs.”* “*So I've not long found out that one of the covid vaccines has only been tested on white people. Which makes no sense as “bame” are the more at risk group and have been neglected once again in research.”*

#### Tweets Mapped to the “Safety” Theme

A large number of people thought a vaccine developed within a year was unsafe, with many “warning” vaccines normally “takes 10 years” to produce. A sense of fear that an “untested,” “experimental” vaccine was being rolled out to the public “lab rats” and “guinea pigs” prompted some to adopt a “wait and see” approach before accepting a vaccine. It appeared the most significant aspect of safety concerns amongst Twitter users was the severity of acute adverse effects (e.g., “skin peeling,” “horror deaths,” “facial paralysis,” and “blood clots”) rather than long-term side effects. A small number of posts expressed safety concerns that delaying the second-dose was dangerous because it was “off-label” and “went against scientific advice.” Vaccinated people took to Twitter to complain of post-vaccine side-effects (e.g., “sore-arm” “flu-like symptoms” “headache”), with a small minority encouraging others to refuse the vaccine as a result.

#### Tweets Mapped to the “Mistrust” Theme

The majority of tweets coded to mistrust surrounded the motivations of the pharmaceutical industry and/or the government. A small number of tweets referenced conspiracy theories such as mass vaccination being a government ploy to weaken the immune system of the “sheep,” or COVID-19 pandemic being deliberately manufactured or deliberately exaggerated by pharmaceutical companies “just for profit” from vaccine administration. Further, users were sceptical of the government's competency to deliver the rollout, citing previous failings during the pandemic, including “test and trace” and “care homes” as reasons not to trust the government. Many did not believe the existence of new “mutant strains” or “new variants,” perceiving them to be “government lies” constructed to cover-up the vaccines “never really worked at all.” This sense of mistrust was further fostered by circulation of a news story reporting that “big pharma” companies were “protected” from “being sued” or accepting any “legal liability” as a result of any adverse events that emerged as a result of the vaccine.

#### Tweets Mapped to the “Ineffective” Theme

There were lots of discussions surrounding vaccines being “less effective than promised” or “ineffective against variants” or how a “second dose delay” would reduce long-term immunity. However, it was difficult to infer if this discussion meant that they would be likely to refuse immunisation as a result of these concerns about effectiveness. The negative sentiment arising from tweets coded to this theme was generated by a sense of despondency that arrival of the vaccines would not resolve the crisis as hoped. Whilst the majority of posts highlighting that you could “still get COVID” despite being vaccinated were using this fact to argue against implantation of the controversial vaccination passports rather than as a reason to refuse vaccination. However, a small number of users were asking “what's the point?” of getting an “ineffective” vaccine that did not prevent COVID transmission, implying that frequent discussion regarding the lack of vaccine effectiveness could reduce uptake.

#### Tweets Mapped to the “Access” Theme

Difficulties relating to accessing vaccines were heavily discussed within our sample of tweets. However, many tweets did not reflect genuine inability to access vaccines, but rather pro-vaccine users upset by people “queue jumping,” particularly those who were not considered “vulnerable.” A key issue consistently emerging within tweets coded to access was difficulty booking an appointment with frustration expressed at how “time-consuming” or “impossible” the process was. In particular, users reported making numerous attempts to secure an appointment using the NHS online system, with some remarking that it was “harder than getting Glastonbury tickets.” A small number of tweets reported how vaccine-related “scams” and “fake texts” had made them doubt NHS text message reminder notifications, resulting in missed appointments.

#### Tweets Mapped to the “Complacency” Theme

Some users' tweets expressed a complacent attitude towards the need for vaccines. Tweets using phrases like “what's the point?” or “no need” were often posted in response to vaccine-related news articles being shared. Some people believed that only the vulnerable needed to be vaccinated and that mass-vaccination had been an “over-reaction.” Complacency detected amongst some users emerged as declarations that they were “over COVID,” and just wanted a return to normality.

#### Tweets Considered Misinformation

Tweets classified as misinformation were heavily biased towards safety, mistrust, and efficacy concerns. A large volume of tweets were identified as misinformation either in the form of a lack of user knowledge or posts from so called “anti-vaxxers” deliberately attempting to spread rumours to discourage vaccination. A number of individuals did not believe the coronavirus pandemic was real and many posts referenced anecdotal “evidence” of how dangerous the vaccines were such as: “HORROR! as 27 die suddenly after taking Pfizer jab” or “man left in agony as skin peels off hours after getting Astrazeneca vaccine.” Although most tweets tagged as misinformation appeared to be coming from “anti-vaxxers,” or vaccine hesitant individuals, a small minority of users were posting inaccurate information to encourage vaccination. For example, tweets were identified that shared false statistics on the relative risk of blood clots after taking the AstraZeneca vaccine compared to taking the contraceptive pill. Social media is a vital tool for disseminating health information; however, the high proportion of tweets in our sample coded as misinformation also highlights the potential concerns. Fostering online communities who refute scientific advice and instead make healthcare decisions based on the false online information creates substantial public health risk.

## Discussion

The purpose of this study was to further investigate the application of NLP techniques as a means of gathering evidence from unstructured, soft-intelligence data sources and assess the utility of this to inform public health research or decision making. As a case study we deployed a commercial, AI-driven NLP platform to leverage insights from Twitter data, with the aim of rapidly identifying key barriers to COVID-19 vaccination uptake amongst users in London.

Throughout the analysis period (30 November 2020 to 15 August 2021), 91,473 Tweets referencing COVID-19 vaccines were posted from London Twitter accounts. The specialist text analytics platform we deployed assigned all of the collected tweets as having positive, neutral, or negative sentiment. The platform utilised machine learning to automatically extract the tweets from the 12 most common topic clusters underpinned with negative sentiment to generate a sample corpus of 913 tweets to perform qualitative analysis.

Results from our qualitative analysis highlighted the polarising views amongst different users in the online vaccine discourse. We identified concerns over vaccine safety, and mistrust towards the government or pharmaceutical companies to be the two major themes relating to vaccine hesitancy. We also identified numerous tweets that contained and reported misinformation. This further highlights that whilst social media can be a powerful means to disseminate useful health-related information, it can equally be used to spread false and potentially harmful information, thus posing a public health risk.

Across all vaccine hesitancy themes the main issues preventing uptake were:

- Concern that vaccines developed so quickly must be experimental and fears that inadequate testing could result in adverse side effects amongst those who take it.
- Beliefs that pandemic was being falsely reported by the media and accusations levelled at the government of fabricating data to coerce mass vaccination.
- Anger and anxiety that pharmaceutical profits were being prioritised before population safety.
- Scepticism regarding vaccine efficacy.
- Belief that only those who were old or vulnerable needed to be vaccinated.
- Access issues. In particular, difficulty using the online booking system.

Our approach identified similar themes underpinning vaccine hesitancy compared to previous longitudinal surveys assessing vaccine hesitancy in the UK (32). However, by utilising the automated topic and sentiment clustering capabilities of the platform we deployed, a case can be made that the findings were acquired using less time and resources on the part of the researchers. Additionally, this case study used an off-the-shelf platform rather than an internally developed, bespoke, AI tool, making this technology more accessible to the researchers. The mixed-method approach adopted allowed for a more nuanced analysis to be undertaken, using a more robust established methodology. Despite the need for human input, the overall resource required to produce this research was reduced considerably through using the NLP tool.

This study contributes to a growing body of work investigating Twitter as a source of soft-intelligence, which can be used to capture real-time public insights, attitudes, and emerging trends concerning a particular health issue (15, 18, 36). Most of this existing research has largely been conducted through qualitative analysis of a small sample of tweets that are selected randomly from within a large dataset (40, 41). Here we present a case study that helps demonstrate the advantages of using a specialist AI-driven NLP tool that can be tailored to generate a corpus of tweets capturing the most common negative topics of discussion rapidly and automatically within a large dataset as a basis for further contextual evaluation of the topics.

Using machine learning to generate a high-quality sample set of the most relevant tweets enabled a quicker and more focused qualitative analysis. It removed the need for a researcher to manually sift through many 100s and 1000s of irrelevant posts, thereby increasing the efficiency and reducing the time and resources necessary to answer the research question.

This is a small case study that demonstrates the feasibility of AI tools to efficiently compile a corpus of relevant data to be analysed more robustly using established methods. Like other research of these methods, it shows that this is a promising methodology that has the potential to become a valuable addition to a well-established portfolio of evidence synthesis methods. As with any new methodology, further research is still required, with an initial focus on the issues of generalisability and bias that the use of these types of data and tools may bring. However, our case study and other work in this field suggests that there is a place for these analysis tools, alongside more established methods of evidence synthesis, when addressing some public health research questions.

## Limitations

The data that is available from social media is a sub-population and hence raises questions regarding generalisability. For our case study, the demographic of London Twitter users is not representative of the demographic of the London population and may differ in terms of age, gender, and socioeconomic status. Since it is not possible to collect such demographic data while maintaining the users' anonymity, this is a limitation faced when using this social media site as a source of soft intelligence. More work is required to fully explore this; currently it is unclear whether the issue is as significant as perceived.

As with many AI NLP solutions, the nuance of language can lead to odd results. In this example, the platform struggled to ascertain the true context of certain tweets. For example, based on the platform's automatic topic and sentiment clustering, it would have appeared that vaccine passports were amongst the most significant barriers to vaccination. In actuality, most tweets discussing vaccine passports supported the vaccine but strongly opposed vaccine passport introduction. We tried to overcome this limitation by taking a mixed methods approach and incorporating some element of manual screening to ensure the validity of results. However, as with all research involving qualitative data analysis, there is a need to ensure that there is a consensus between reviewers and consistency in approach. Given that our search strategy generated almost 100,000 tweets, we decided to limit topic and qualitative analysis to negative sentiment tweets. Whilst tweets expressing vaccine hesitancy were most likely to have been classified as negative, there may have been some classified as neutral and therefore not included for analysis. We would suggest based on this, that it may be of benefit in future work to review samples of the tweets excluded at the neutral sentiment and topic clustering stages to further assess the viability of the platform (see Figure 1). There may be limitations to the methods we used for the case study itself, for example we may have expanded our search terms to include more informal words for vaccines such as “jab.” However, these are all subjective decisions that like any evidence synthesis research should be determined *a priori*, transparently report and justified. Given the focus of the paper these limitations are not discussed fully.

## Future Work

This case study encapsulated a 6-month period of the pandemic. To truly assess the use of the platform to provide a rapid analysis of public sentiment in the public health space, it may be of worth capturing a smaller time period with a broader geographical range. Additionally, this tool could be deployed on other public forums such as MumsNet and Redditt to assess vaccine hesitancy amongst specific population groups who have lower vaccination rates. Finally, work to explore how this kind of analysis might inform and evolve existing mixed methods approaches, including those leveraging established behavioural models (e.g., BCW and COM-B) (42, 43). This could help to develop targeted intervention strategies, that have maximum impact on vaccine uptake.

We believe that this work demonstrates the utility for off-the-shelf NLP tools to leverage insights from social media data to support public health research as part of a mixed-methods approach or during times of crisis when rapid and reactive public health engagement is needed.

## Data Availability Statement

The raw data supporting the conclusions of this article will be made available by the authors, without undue reservation.

## Author Contributions

KL and CM contributed to conception and design of the study. KL and RG performed the mapping and qualitative document analysis. KL wrote the first draft of the manuscript. KL, CM, RG, and DC wrote sections of the manuscript. All authors contributed to manuscript revision, read, and approved the submitted version.

## Funding

This project was funded by the National Institute for Health Research (NIHR) (HSRIC-2015-1009/Innovation Observatory). The views expressed are those of the author and not necessarily those of the NHS, the NIHR or the Department of Health.

## Author Disclaimer

The views expressed are those of the author(s) and not necessarily those of the NIHR or the Department of Health and Social Care.

## Conflict of Interest

The authors declare that the research was conducted in the absence of any commercial or financial relationships that could be construed as a potential conflict of interest.

## Publisher's Note

All claims expressed in this article are solely those of the authors and do not necessarily represent those of their affiliated organizations, or those of the publisher, the editors and the reviewers. Any product that may be evaluated in this article, or claim that may be made by its manufacturer, is not guaranteed or endorsed by the publisher.

## Abbreviations

AI: Artificial Intelligence
BCW: Behaviour Change wheel
COM-B: Capability and Behaviour Model
MHRA: Medicines and Healthcare Regulatory Agency
NIHR: National Institute for Health Research
NLP: Natural Language Processing
SAGE: Strategic Advisory Group of Experts
WHO: World Health Organisation.
